# Respiratory and locomotor muscle blood flow measurements using near-infrared spectroscopy and indocyanine green dye in health and disease

**DOI:** 10.1177/14799731241246802

**Published:** 2024-04-08

**Authors:** Dimitrios Megaritis, Carlos Echevarria, Ioannis Vogiatzis

**Affiliations:** 1 Department of Sport, Exercise and Rehabilitation, Faculty of Health and Life Sciences, Northumbria University Newcastle, Newcastle Upon Tyne, UK; 2 Respiratory department 5983 , The Newcastle Upon Tyne Hospitals NHS Foundation Trust, Newcastle Upon Tyne, UK; 3 ICM, Newcastle University, Newcastle Upon Tyne, UK

**Keywords:** Muscle blood flow, near-infrared spectroscopy, chronic obstructive pulmonary disease

## Abstract

Measuring respiratory and locomotor muscle blood flow during exercise is pivotal for understanding the factors limiting exercise tolerance in health and disease. Traditional methods to measure muscle blood flow present limitations for exercise testing. This article reviews a method utilising near-infrared spectroscopy (NIRS) in combination with the light-absorbing tracer indocyanine green dye (ICG) to simultaneously assess respiratory and locomotor muscle blood flow during exercise in health and disease. NIRS provides high spatiotemporal resolution and can detect chromophore concentrations. Intravenously administered ICG binds to albumin and undergoes rapid metabolism, making it suitable for repeated measurements. NIRS-ICG allows calculation of local muscle blood flow based on the rate of ICG accumulation in the muscle over time. Studies presented in this review provide evidence of the technical and clinical validity of the NIRS-ICG method in quantifying respiratory and locomotor muscle blood flow. Over the past decade, use of this method during exercise has provided insights into respiratory and locomotor muscle blood flow competition theory and the effect of ergogenic aids and pharmacological agents on local muscle blood flow distribution in COPD. Originally, arterial blood sampling was required via a photodensitometer, though the method has subsequently been adapted to provide a local muscle blood flow index using venous cannulation. In summary, the significance of the NIRS-ICG method is that it provides a minimally invasive tool to simultaneously assess respiratory and locomotor muscle blood flow at rest and during exercise in health and disease to better appreciate the impact of ergogenic aids or pharmacological treatments.

## Introduction

The respiratory muscles ensure that one can achieve an adequate level of ventilation, given prevailing metabolic demands, thereby ensuring adequate oxygen supply to exercising muscles and removal of carbon dioxide. Respiratory and peripheral muscle dysfunction is well documented in patients with chronic respiratory diseases.^1–3^ Measuring tissue blood flow is pivotal for understanding the pathophysiology and exercise tolerance in both health and disease.

An important question revolves around the (re)distribution of blood flow between the respiratory and the locomotor muscles during physical exertion,^4^ particularly in pathophysiological states seen in conditions such as COPD. Exercise programmes and/or pharmacological agents that improve muscle blood flow may improve function in COPD patients, especially in those with insufficient or diminished blood flow during normal breathing or exertion.^5^ Consequently, measuring respiratory muscle blood flow may play a crucial role in understanding the patterns of blood flow distribution acutely during administration of pharmacological agents, ergogenic aids and during exertion, both in healthy individuals and patients with COPD.

Previous approaches to measuring tissue blood flow in humans were invasive and had several limitations. The conventional tracer methods require the arterial injection of an indicator or tracer such as ^133^Xenon into the limb of the muscle and venous measurement of the rate of wash-out.^6^ As well as requiring arterial and muscle (venous) cannulation, it does not distinguish between other tissues of the limb, such as active and inactive muscles, skin, or adipose tissue.^6,7^ Another invasive approach measures the timed volume collection of blood droplets from the left inferior phrenic vein via a 100 cm long catheter.^5^ Venous plethysmography is a non-invasive method where venous outflow from a limb is temporarily occluded, thus any increase in limb volume is attributed to arterial inflow. However, this method evaluates the overall blood flow of the limb rather than focusing on a specific muscle group.^8^ These traditional invasive approaches are unsuitable for exercise testing due to the need for catheterisation of the inferior phrenic vein, which is highly intrusive and necessitates iatrogenic sedation.

Moreover, assessing muscle blood flow to multiple respiratory muscles during exercise poses challenges due to the intricate anatomical arrangement or the respiratory muscles, the complex network of arterial and venous vessels, and the diverse variations in muscular recruitment that occurs within respiratory muscles.^9–11^

To overcome these limitations in measuring tissue blood flow, a more versatile method was imperative for quantifying blood flow in human subjects. Consequently, this review focuses on studies that have employed a novel technique utilising near-infrared spectroscopy (NIRS) in conjunction with the light-absorbing tracer indocyanine green dye (ICG) to measure local muscle blood flow in patients with COPD. This includes studies that measured respiratory or locomotor muscle blood flow and used medication and ergogenic aids during exercise.

### Measuring muscle blood flow using the novel NIRS-ICG method

NIRS is a non-invasive modality which includes near-infrared light in the spectrum of wavelengths from 700 to 1000 nm to non-invasively measure dynamic changes in tissue oxygenation. NIRS can easily pass through tissues and directly measuring the concentration (and changes in concentration) of chromophores such as oxyhaemoglobin and deoxyhaemoglobin.^12^ It should be noted that influence of myoglobin oxygenation to the overall NIRS signal is relatively small.^13^ Since the early years of development (1970s), NIRS has undergone significant advancements,^10^ and has several advantages over older approaches, including non-invasiveness, minimal susceptibility to movement artifacts, and high spatiotemporal resolution.^14^

Indocyanine green dye (ICG) is a water-soluble tricarbocyanine with peak absorption in the near-infrared range of 800 nm within human blood. It binds rapidly to albumin in the blood, and remains in the vascular compartment, making it good tracer for measuring blood flow and perfusion. By shining near-infrared light into the tissue of interest the amount of light absorbed by ICG can be measured, allowing inferences about the blood flow and tissue perfusion. In the NIRS-ICG method, ICG is transcutaneously detected by measuring light attenuation at specific wavelengths, including 775 nm, 813 nm, 850 nm, and 913 nm. The analyses are conducted using an algorithm that incorporates the modified Beer-Lambert law.^15^ It is noteworthy that extracting ICG signals requires careful consideration of these specific wavelengths, and the process involves algorithms to accurately quantify blood flow and perfusion. It should be noted that not all commercially available NIRS systems have the capacity to detect ICG. While we have aimed to make this review accessible to a broader clinical and research audience, we acknowledge the technical intricacies involved in implementing this technique, particularly regarding the necessity for precise wavelength measurements.^15^

ICG has been used to measure cardiac output, as well as limb muscle blood flow when combined with photodensitometry.^16^ Over the past 20 years, use of NIRS with the light absorbing ICG has been employed to assess locomotor muscle blood flow based on the application of Fick’s principle,^17^ incorporating the calculation of the rate of accumulation of ICG in the tissue over time. Several studies have utilised the aforementioned methodology to examine various tissues, such as those related to respiratory and locomotor muscles,^18–22^ the brain,^23–26^ and connective tissues.^17^ Furthermore, NIRS has proven valuable in the clinical setting for assessment of circulatory and metabolic abnormalities in a wide range of disease entities.^27–30^ Table 1 summarises the use of NIRS-ICG during different modalities, muscles and populations.

**Table 1.** Use of NIRS-ICG during different modalities, muscles and populations.

Study	Population	Modality	Muscles of interest
Vogiatzis et al., (2010)^19^	COPD	Cycling exercise	Intercostal and vastus lateralis
Louvaris et al., (2021)^43^	COPD	Cycling exercise	Intercostal, scalene, rectus abdominis, and vastus lateralis
Vogiatzis et al., (2011)^48^	COPD	Normoxia vs normoxic heliox breathing during cycling exercise	Intercostal and vastus lateralis
Louvaris et al., (2014)^49^	COPD	Normoxia vs hyperoxia vs normoxic heliox breathing during cycling exercise	Intercostal, abdominal, and vastus lateralis
Berton et al., (2019)^50^	COPD	Post-bronchodilator vs placebo, cycling exercise	Quadriceps
Ribeiro et al., (2021)^51^	COPD	Sympathoexcitatory manoeuvre	Intercostal and vastus lateralis
Louvaris et al., (2019)^52^	Ventilated patients	Spontaneous breathing trials	Scalene and upper rectus abdominis
Boushel et al., (2002)^35^	Healthy	Prostaglandins, nitric oxide and endothelial-derived hyperpolarizing factors	Quadriceps
Guenette et al., (2008)^15^	Healthy	Rest and isocapnic hyperpnoea	Intercostal and vastus lateralis
Vogiatzis et al., (2008)^37^	Healthy (athletes)	Hypoxia vs normoxia vs hyperoxia, cycling exercise	Intercostal and vastus lateralis
Vogiatzis et al., (2009)^36^	Healthy (athletes)	Cycling exercise	Intercostal and vastus lateralis
Habazettl et al., (2010)^7^	Healthy (athletes)	Cycling exercise	Intercostal and vastus lateralis
Boushel et al., (2000)^17^	Healthy	Plantar flexion	Achilles’ peritendinous tissue, calf muscle

The muscle blood flow assessment method requires arterial canulation and sampling of arterial blood through a photodensitometer.^7^ Thus, the arterial ICG curve in combination with the NIRS derived data is used to calculate muscle blood flow locally. Following an intravenous injection, ICG predominantly binds to albumin^31^ and undergoes rapid metabolism by hepatic parenchymal cells, rendering it suitable and safe for repeated blood flow measurements. Local muscle blood flow is calculated based on the rate of tissue ICG accumulation over time, which is measured by NIRS, in accordance with the Saperstein principle.^32^ Consequently, within any given time interval prior to reaching the peak tissue accumulation of the tracer, the tissue receives the same proportion of the ICG bolus as quantified in arterial blood. Two distinct time points within the initial half of the curve are employed to calculate flow, and the average value represents ICG accumulation. The total blood flow is derived using the following equation:

$$blood\;flow\;\left(ml\cdot 100{ml}^{-1}\cdot \min^{-1}\right)=\frac{k\cdot {\left[ICG\right]}_{m}\cdot t}{\int_{0}^{t}{\left[ICG\right]}_{a}dt}$$

where k is a constant representing the molecular weight of ICG for the conversion of ICG in moles to grams per litre; [ICG]_m_ is the accumulation of ICG in tissue over time t expressed in micromoles; and $\int_{0}^{t}{\left[ICG\right]}_{\alpha }dt$ is the time integral of the arterial ICG concentration expressed in milligrams per litre.^6^

**Figure 1.**
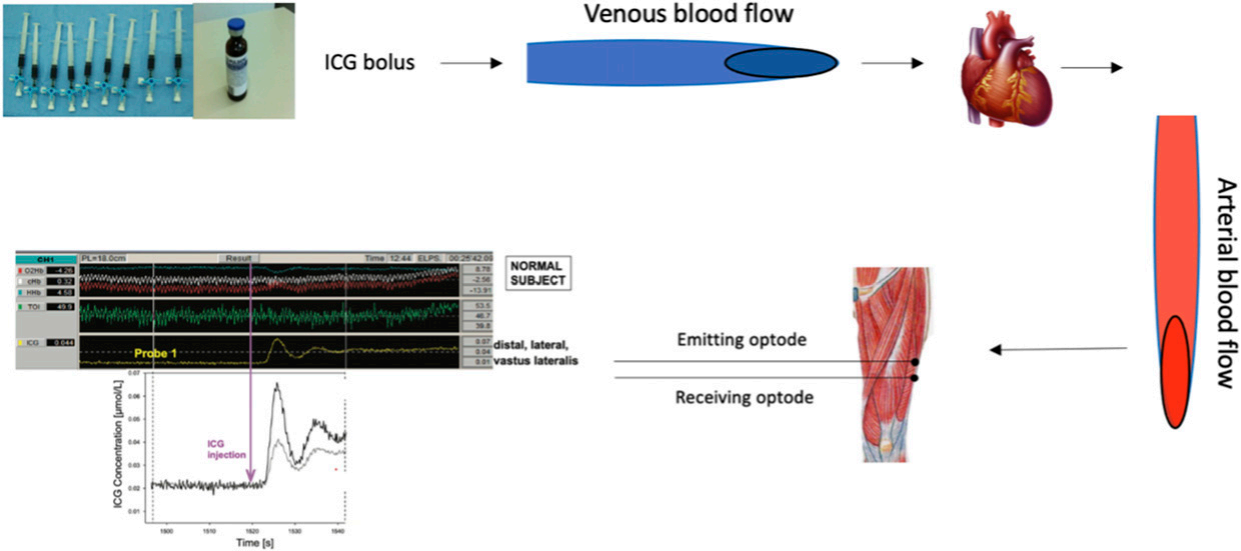
Schematic representation of the NIRS-ICG technique. From top left to bottom: an intravenous bolus of ICG is administered intravenously, it passes through the heart and lungs through the venous circulation, then via the arterial circulation it enters the systemic circulation and microcirculation. The NIRS optodes which are positioned over the vastus lateralis muscle, detect the ICG concentration. Extinction coefficients in a matrix operation are employed, and the ICG curve is isolated. The circles represent the NIRS optodes positioned over the muscle tissue. Output from one NIRS channel, showing the indocyanine green (ICG) dye signal (yellow) following intravenous ICG injection marked by the purple arrow. Green trace [tissue oxygenation index (TOI)] is the oxygenation signal. (Output from NIRS channels was reproduced from Vogiatzis I. et al., 2015; Journal of Applied Physiology, 118 (6), p783-793).

Following injection of the ICG bolus into the venous blood, the tracer is circulated through the circulatory system, reaching the right side of the heart and subsequently the lungs, thus entering the arterial circulation (Figure 1). Then, an external pump withdraws arterial blood while the photodensitometer records the concentration of ICG within the arterial blood. Additionally, within the muscle tissue microcirculation downstream, the accumulation of ICG is detected by measuring the attenuation of light using NIRS.^17^ This technique has the advantage of being able to quantify muscle blood flow (in ml∙100 mL tissue^−1^∙min^−1^) while providing simultaneous measurement of cardiac output.^6^ It is essential to note that once ICG is introduced into the venous circulation, it is pumped via the heart to the rest of the body. Therefore, the method involves strategically placing optodes on muscles of interest to measure the absorbance of ICG. Thus, the NIRS-ICG method allows simultaneous assessment of regional blood flow in different sites of the same muscle or across multiple muscle groups.

### Blood flow index (BFI) and the adapted NIRS-ICG method

**Figure 2.**
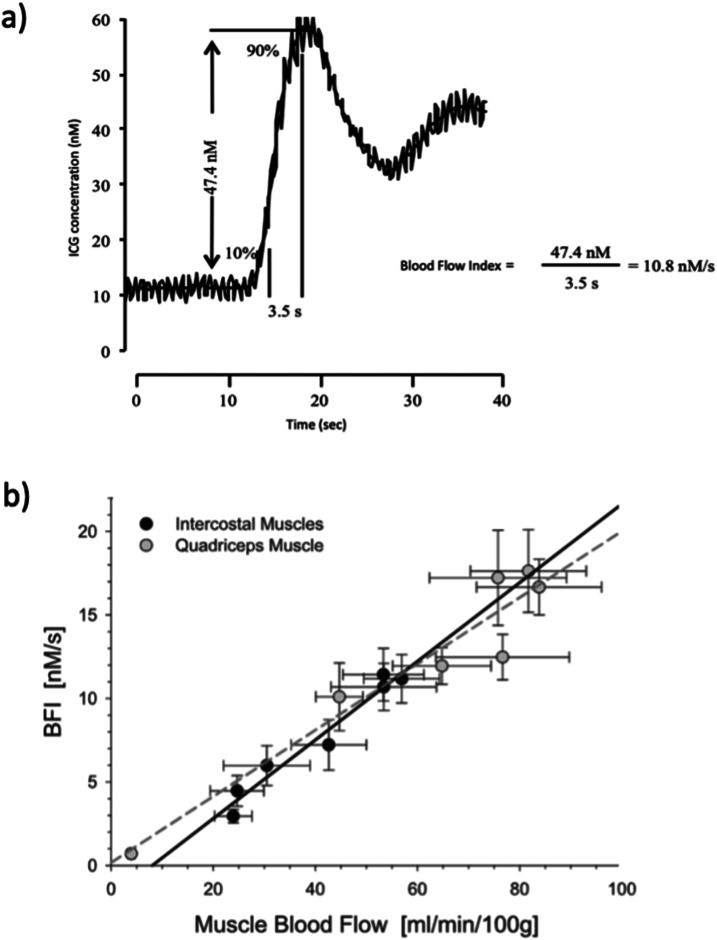
(a) Example of Blood Flow Index calculation; (b) Regression analyses of Blood FIow Index (BFI) versus local muscle blood flow for intercostal (filed symbols, black line) and quadriceps (open symbols, grey line) muscles; r = 0.98 for intercostal and 0.96 for quadriceps muscle. *p* < .001 for both muscles. (Reproduced from original data from Guenette et al., 2008; Journal of Applied Physiology, 104 (4), p1202-1210).

The original NIRS and ICG method described above is considered invasive, given that it requires arterial cannulation and the continuous withdrawal of blood using a photodensitometer for several seconds after injecting the ICG. Arterial cannulation carries potential risks such as bleeding, vascular perforation, vascular insufficiency, and nerve injury.^33^ It also requires sophisticated equipment to maintain a constant blood withdrawal rate through a photodensitometer to measure ICG concentration. Therefore, the original NIRS-ICG technique may not be applicable in all situations. An alternative approach to derive a blood flow index (BFI) has been proposed to calculate tissue perfusion from NIRS data alone, without the use of arterial canulation.^7^ The BFI is calculated by dividing the peak concentration of ICG by the rise time from 10% to 90% of the peak (Figure 2(a)).^7^ Importantly, the BFI is derived solely from the NIRS-ICG curve measured through the skin and does not require arterial blood photodensitometry analysis. The only invasive aspect of this technique remains the venous cannulation for injecting the ICG tracer. Therefore, calculation of the BFI provides a relative index of local muscle blood flow, since the absolute blood flow cannot be directly determined without assessing arterial ICG concentration (through a photodensitometer). In patients with COPD, the BFI method demonstrates high convergent validity as well as, revealing strong agreement with MBF during rest and across various exercise intensities sustained at 25%, 50%, 75%, and 100% of peak work rate.^34^ The significant associations were observed for both intercostal and vastus lateralis muscles, emphasising the convergent validity of BFI in COPD patients.^34^

## Validation of the NIRS-ICG method

The NIRS-ICG technique encompassing arterial blood analysis though a photodensitometer has been utilised and validated for assessing blood flow in respiratory and locomotor muscles during exercise, comparing it to established methods such as dye dilution, ^133^Xe washout, and magnetic resonance imaging in healthy individuals and patients with COPD.^15,17,19,35,36^ The findings consistently indicate that this technique is valid and accurate in quantifying local blood flow in both respiratory and locomotor muscles during exercise. For instance, Boushel et al. ^17^ employed the NIRS-ICG technique to measure blood flow in the calf and Achilles tendon regions during plantar flexion exercise in healthy participants. The results revealed a linear increase in tissue ICG accumulation in these regions as workload increased, and a strong correlation was found between NIRS-ICG measurements and ^133^Xe washout and magnetic resonance imaging methods during exercise.^17^ Similarly, the same group of researchers conducted a study in healthy subjects, quantifying blood flow in the vastus lateralis and vastus medialis muscles during dynamic knee extension exercise.^35^ A linear increase in muscle blood flow was observed with increasing workload, while inhibition of muscle vasodilation factors during exercise led to a decrease in muscle blood flow. This further confirms the validity and sensitivity of the NIRS-ICG technique for assessing locomotor muscle blood flow during exercise.^35^

**Figure 3.**
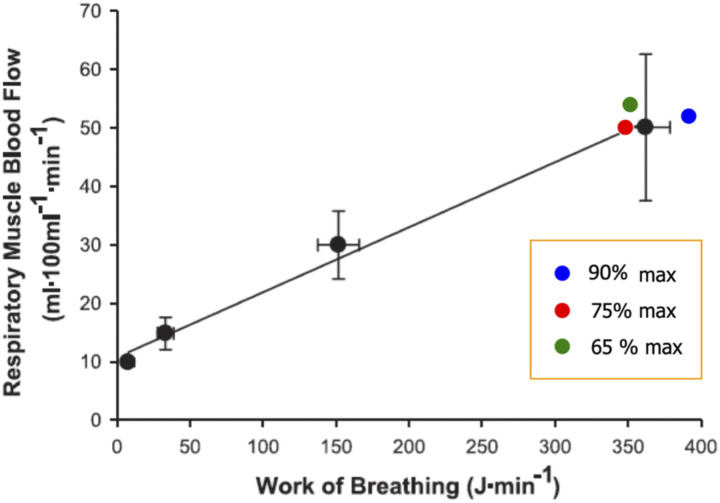
Association between intercostal muscle blood flow and the rate of respiratory muscle work across different levels of minute ventilation during resting isocapnic hyperpnoea (black symbols) and graded incremental exercise (coloured symbols) in healthy subjects. (Reproduced from original data from Vogiatzis et al., 2009; J Physiol, 587 (14), p3665-3677).

Guenette and colleagues were the first to quantify respiratory muscle blood flow in athletes during resting isocapnic hyperpnea at different fractions of maximum minute ventilation using the NIRS-ICG method.^15^ In the aforementioned study, NIRS optodes were placed on the seventh intercostal space at the apposition of the costal diaphragm encompassing the internal and external intercostal muscles, while oesophageal and gastric pressures were concurrently measured to calculate the rate of respiratory muscle work. A subsequent study in the same healthy subjects during incremental cycling confirmed the accuracy of intercostal muscle blood flow relative to the rate of respiratory muscle work (Figure 3).^37^

**Figure 4.**
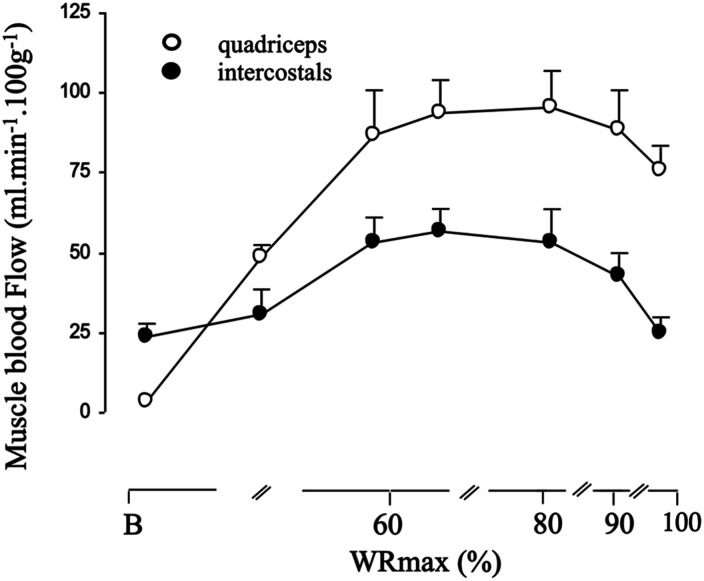
Intercostal and quadriceps muscle blood flow at baseline (B) and across different levels of cycling exercise to the limit of tolerance. Data obtained from athletes. Values are means ± SEM. (Reproduced from original data from Habazettl et al., 2010; Journal of Applied Physiology, 108 (4), p962-967).

Additionally, Vogiatzis et al. employed the same NIRS-ICG technique to simultaneously measure intercostal and quadriceps muscle blood flow during graded incremental exercise in athletes.^36^ In these studies measurements were conducted during resting isocapnic hyperpnea and various work intensities up to peak cycling exercise.^36^ The results indicated that intercostal and quadriceps muscle blood flow increased linearly up to 80% of peak work rate. At higher intensities both intercostal and quadriceps muscle blood flow started to fall (modestly) at higher work rates (Figure 4). Cardiac output also rose as expected from rest to 80% of peak exercise, but then essentially plateaued over the upper 20% of exercise. These results were interpreted as reflecting the inability of the circulatory system to preserve perfusion to these muscle groups during maximal levels of exercise.^36^

Additionally, the NIRS-ICG technique using the BFI method has exhibited high convergent validity. Traditionally, it has been used for assessing cerebral blood flow^38–41^ and recently was validated for measuring skeletal muscle perfusion.^7,15^ In a retrospective analysis conducted by Habazettl and colleagues, BFI values from the vastus lateralis muscle and the 7th intercostal space were compared to absolute muscle blood flow measured using the NIRS-ICG method (incorporating arterial blood analysis though a photodensitometer) during cycling exercise at different intensities in healthy subjects.^7^ The results showed a strong agreement between the two methods for both the respiratory and quadriceps muscles (Figure 2(b)). Additionally, a study by Guenette and colleagues^15^ extended these findings by investigating whether the BFI can assess blood flow to the respiratory muscles by designing a prospective experimental design. BFI measurements were taken from the intercostal and sternocleidomastoid muscles during resting isocapnic hyperpnea, while simultaneously measuring electroneurography and rate of respiratory muscle work.^15^ The results agreed with the ones from Habazettl et al.,^7^ as the BFI showed a significant correlation with the rate of respiratory muscle work and electromyography data for the respiratory and locomotor muscles, respectively. Add here convergent validity in COPD. These findings suggest that using the BFI closely reflects respiratory muscle blood flow across a wide range of exercise intensities and levels of minute ventilation, offering a less invasive and technically demanding method for measuring muscle perfusion during exercise.

### Respiratory and locomotor muscle blood flow regulation during exercise in COPD

**Figure 5.**
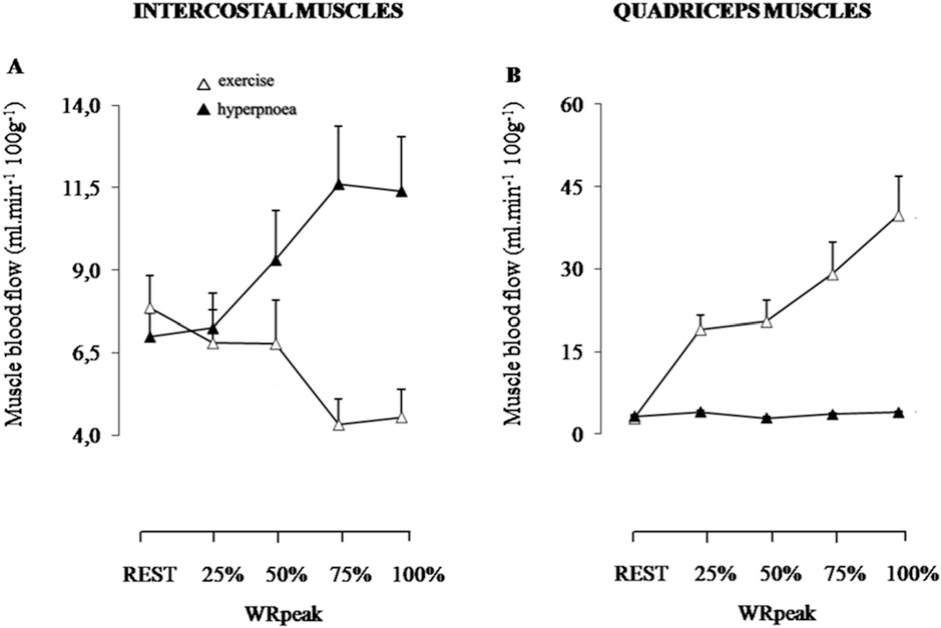
Intercostal and quadriceps muscle blood flow during exercise (open triangles) and resting isocapnic hyperpnoea (closed triangles) in patients with COPD.(Reprinted with permission of the American Thoracic Society. Copyright © 2024 American Thoracic Society. All rights reserved. Vogiatzis et al., 2010; Intercostal muscle blood flow limitation during exercise in chronic obstructive pulmonary disease. Am J Respir Crit Care Med, 182(9), p1105-1113. The American Journal of Respiratory and Critical Care Medicine is an official journal of the American Thoracic Society).

In patients with COPD, the progressive development of expiratory flow limitation during exercise increases the rate of respiratory muscle work and the metabolic requirement of the respiratory muscles.^1^ The hypothesis of whether a competition for blood flow occurs between the locomotor and respiratory muscles during exercise has been tested using the NIRS-ICG technique in patients with COPD.^19^ In this study authors measured quadriceps and intercostal muscle blood flow and cardiac output during resting hyperpnoea and from rest to peak exercise in five steps in 10 COPD patients (FEV_1_: 51% predicted).^19^ Hyperpnoea results again mirrored those of Guenette et al.^15^ with an exponential rise in intercostal muscle blood flow from rest to peak isocapnic ventilation (Figure 5). Exercise results showed cardiac output increasing until about 75% of peak work rate and then plateaued. Quadriceps muscle blood flow increased linearly with the increase in work rate (Figure 5). Unexpectedly, intercostal muscle blood flow fell progressively from rest to peak exercise (Figure 5). In fact, intercostal muscle blood flow began to fall even during light exercise, and as cardiac output was rising. As cardiac output plateaued, a greater fall in intercostal flow now occurred. There was no evidence of redistribution of blood from legs to respiratory muscles; if anything one could argue the opposite from the reduction in intercostal muscle blood flow. However, with intercostal muscle blood flow falling even as cardiac output was increasing, it did not seem that a limited cardiac output was the cause of intercostal muscle blood flow reduction. These findings were interpreted as challenging the blood flow redistribution theory.^42^ The above findings were confirmed by a recent study assessing several non-diaphragmatic respiratory muscles.^43^ In a recent study involving COPD patients, the blood flow index (BFI) was assessed across several respiratory muscles, including intercostal, rectus abdominis, and scalene muscles, during loaded breathing and hyperpnoea.^44^ The results indicated that muscle perfusion significantly increased from rest in the scalene muscle during hyperpnoea compared to loaded breathing. However, no statistically significant differences were observed in the remaining muscles between the two conditions.^44^

### Effect of ergogenic aids and pharmacological agents on muscle blood flow in COPD

**Figure 6.**
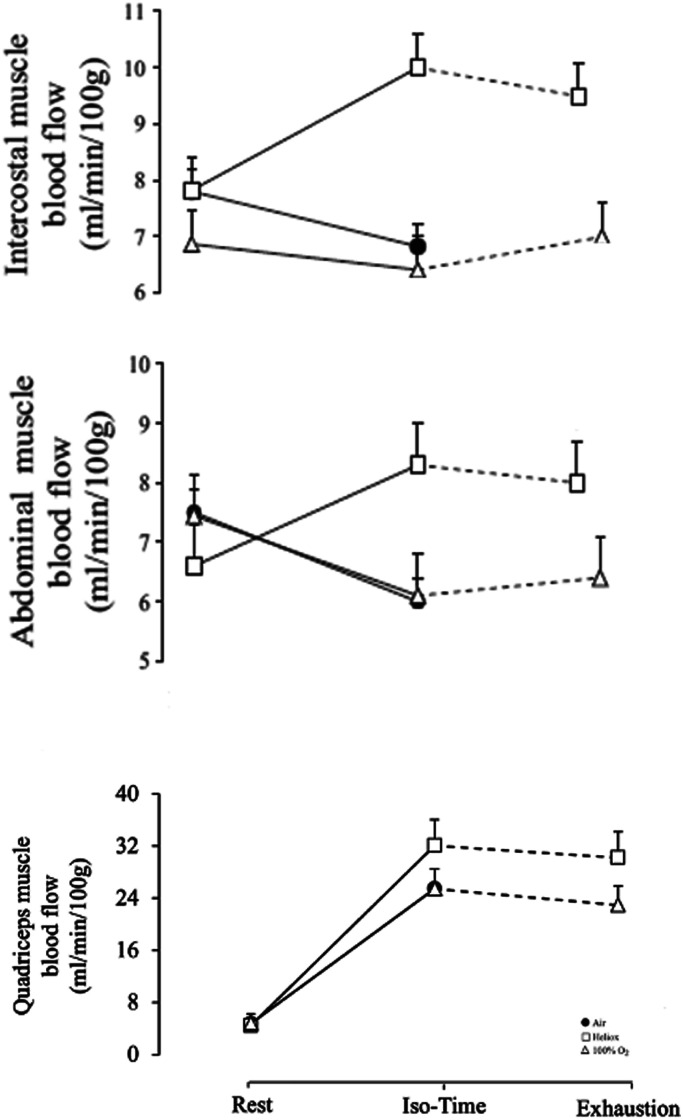
Intercostal, abdominal and quadriceps muscle blood flow during exercise; open triangles indicate hyperoxia, closed circles indicate normoxia and open squares indicate heliox, in COPD patients. Isotime: the time point where exercise in normoxia was terminated. (Reproduced from original data from Louvaris et al., 2014; Journal of Applied Physiology, 117 (3), p267-276).

In healthy individuals, there is evidence suggesting that reducing the rate of respiratory muscle work might be associated with an increase in locomotor muscle blood flow.^45–47^ In patients with COPD, it has been hypothesised that lessening the rate of respiratory muscle work via administration of ergogenic aids such as heliox or oxygen, might lead to blood flow redistribution from the respiratory to the locomotor muscles. Decreased rate of respiratory muscle work has been associated with decreased ‘respiratory muscle metaboreflex’ triggering, thus causing reduced sympathetic efferent discharge to the leg muscles, possibly allowing for improved leg muscle blood flow.^47^ To test this hypothesis studies employing the NIRS-ICG method in COPD simultaneously assessed blood flow in both respiratory and locomotor muscles during constant-load exercise in three conditions: normoxia, hyperoxia and during heliox supplementation^48,49^ (Figure 6).

In these studies authors measured blood flow in the intercostal and locomotor muscles during exercise with and without heliox (21% oxygen/helium). The exercise was kept constant at the same power, heliox was used as it can reduce the rate of respiratory muscle work due to its low density and low resistance to flow. The hypothesis was that if heliox breathing leads to reduced intercostal muscle blood flow, but increased blood flow in the locomotor muscles, this would indicate a redistribution of blood flow from intercostal muscles to locomotor muscles. Authors measured cardiac output, quadriceps, abdominal and intercostal muscle blood flow during constant load exercise to the limit of tolerance – during air and heliox breathing. Both quadriceps and respiratory muscle blood flow were higher with heliox than with air at isotime (time corresponding to exercise termination in normoxia). Cardiac output was slightly higher with heliox at both work rates. Thus, reducing the rate of respiratory muscle work did not change relative quadriceps or respiratory muscle blood flow, and there was no redistribution of blood flow between locomotor and intercostal muscles in either direction. These findings contradict the blood flow redistribution theory.^48,49^

Additionally, administration of oxygen did not lead to altered locomotor, or respiratory muscle blood flow compared to normoxia. Whilst previous reports suggested that reducing the rate of respiratory muscle work is associated with a redistribution of blood flow from the respiratory to the locomotor muscles^45,46^ the findings from these studies in COPD^48,49^ do not support this hypothesis as respiratory and locomotor muscle blood flow were not different between normoxia and hyperoxia in COPD^47^ despite a reduction in the rate of respiratory muscle work with oxygen supplementation (Figure 6).

### Effect of bronchodilators on locomotor muscle blood flow measured by the NIRS-ICG technique in COPD

A more recent study assessed the effects of bronchodilators compared to placebo on locomotor muscle blood flow regulation using the NIRS-ICG method to appreciate the mechanisms leading to improved exercise endurance time in COPD.^50^ Dual bronchodilator compared to placebo was not linked to ameliorated locomotor muscle blood flow during exercise.^50^ Thus, the improved endurance time secondary to bronchodilation could not be attributed to enhanced blood flow to the locomotor muscles during cycling exercise in COPD.^50^ Authors concluded that improved exercise tolerance was due to better breathing mechanics following bronchodilator administration.

### Effect of a sympathoexcitatory manoeuvre in microcirculation measured by the NIRS-ICG technique in COPD

Recently a study investigated the microcirculation response to a sympathoexcitatory manoeuvre (Cold Pressor Test) in patients with COPD and age matched individuals using the NIRS-ICG method.^51^ The findings suggest that intercostal and vastus lateralis microcirculation decreased during the sympathoexcitatory manoeuvre only in patients with COPD but not in healthy age matched individuals.^51^ The findings suggest that COPD patients may have impaired microvascular function, especially during sympathetic activation. Implications on muscle function, functional capacity and exercise capacity may be linked to aforementioned impairment in patients with COPD.

### Bedside assessment of respiratory muscle blood flow in mechanically ventilated patients using the NIRS-ICG technique

A recent study evaluated respiratory muscle blood flow during a spontaneous breathing trial, with the objective of investigating the underlying pathophysiology of weaning failure in mechanically ventilated patients.^52^ Louvaris and colleagues found no significant differences in respiratory muscle or prefrontal cortex blood flow between patients who failed weaning and those who succeeded weaning, during the transition from positive pressure ventilation to the spontaneous breathing.^53^ It should be noted that both groups exhibited an increase in respiratory muscle blood flow from the transition between positive pressure ventilation to the spontaneous breathing trial.^53^

## Future applications of the NIRS-ICG method

The NIRS-ICG method could be further explored and utilised in clinical and research settings to better understand the pathophysiology of chronic respiratory diseases and improve patient management. The effects of ergogenic aids and pharmacological agents on respiratory muscle blood flow should continue to be explored using the NIRS-ICG technique in both the bedside setting and during exercise. Understanding the impact of interventions, such as inspiratory muscle training, on respiratory muscle blood flow can provide valuable insights into optimising functional capacity and enhancing respiratory muscle function in patients with chronic respiratory diseases. Additionally, longitudinal study designs utilising the NIRS-ICG method could provide valuable insights into the progression of the pathophysiology of chronic respiratory diseases and the patterns of respiratory muscle flow over time in association to clinically relevant outcomes. Finally, assessing the disease progression and intervention efficacy (i.e., exercise training programs or pharmacological therapies), on respiratory and locomotor muscle perfusion, would enable a better understanding of the relationship with functional and clinical parameters in patients with chronic respiratory diseases.

It is crucial to emphasise that previous evidence supports the safety of ICG as an intravascular tracer in various settings: (i) including gastrointestinal surgery (injected intravenously, in tumors, peritumorally or subcutaneously)^54,55^ (ii) angiography in pediatric populations (injected subcutaneously and intravenously),^56^ and (iii) angiography during pregnancy (injected intravenously).^57^ While we would like to acknowledge some reported adverse events and contraindications, it is important to note that concerns raised, have been addressed in the literature suggesting alternative means of ICG preparation.^58^ The following adverse events have been reported to the best of our knowledge: anaphylactic shock with or without history of allergy to iodides^59,60^ and urticarial reactions.^61^ Researchers in countries where ICG is perceived as high-risk by their Institutional Review Board committees might find it beneficial to engage with their committees and cite this study as a reference when seeking approval for the tracer’s use under exceptional circumstances.

Additionally, the technique’s reliance on a limited number of NIRS machines capable of tracing ICG and the elevated cost of acquiring such specialised NIRS apparatus compared to those not measuring ICG present practical constraints. The successful implementation of the NIRS-ICG method demands skilled personnel. Expertise in the administration of indocyanine green dye, and familiarity with phlebotomy techniques becomes mandatory for comprehensive proficiency in the NIRS-ICG methodology.

## Limitations of the NIRS-ICG method

Despite its advantages, the NIRS-ICG technique has some limitations. The method under review is invasive, requiring venous and/or arterial cannulation, introducing potential risks such as bleeding, vascular perforation, and nerve injury associated with cannulation. Moreover, the cost of purchasing ICG, might pose financial challenges. Lack of availability of ICG in many countries and the strict regulations governing its use in some nations further limits the widespread application of the NIRS-ICS method. Additionally, the technique’s reliance on a limited number of NIRS apparatus capable of tracing ICG and the elevated cost of acquiring such specialised NIRS apparatus compared to those not measuring ICG presents practical constraints. The successful implementation of the NIRS-ICG method demands skilled personnel. Expertise in the administration of indocyanine green dye and with phlebotomy techniques become mandatory for comprehensive proficiency in the NIRS-ICG methodology. These limitations should be considered when evaluating the feasibility and accessibility of the NIRS-ICS technique for assessing muscle blood flow during exercise in diverse clinical and research settings.

## Conclusions

The NIRS-ICG technique has been shown to be accurate for measuring muscle blood flow in health and disease. By employing near-infrared spectroscopy and the light-absorbing tracer indocyanine green dye, this technique allows for the assessment of dynamic changes and quantification of muscle blood flow. The NIRS-ICG method has been validated in various tissues, including respiratory and locomotor muscles, the brain, and connective tissues. Recent study results have questioned the theory of blood flow redistribution between locomotor and respiratory muscles by measuring perfusion in multiple respiratory muscles during exercise. Furthermore, the development of the blood flow index (BFI) method has eliminated the need for arterial cannulation, making the procedure less invasive and more feasible in different settings. NIRS-ICG-derived BFI, representing a minimally invasive method, has indeed allowed studies to focus on primary and secondary respiratory (scalene, parasternal, sternocleidomastoid and abdominal) muscle perfusion responses and patterns, thus providing a more comprehensive resolution on the intriguing topic of muscle blood flow redistribution during exercise in COPD. Studies using the NIRS-ICG method have shed light on the effects of physical exertion, ergogenic aids, and pharmacological agents on muscle blood flow in patients with chronic respiratory diseases, leading to a better understanding of the underlying pathophysiology. The NIRS-ICG method holds great potential for furthering our understanding on tissue blood flow regulation and its implications for various physiological and pathological conditions.
